# Differential Properties of Venom Peptides and Proteins in Solitary *vs.* Social Hunting Wasps

**DOI:** 10.3390/toxins8020032

**Published:** 2016-01-22

**Authors:** Si Hyeock Lee, Ji Hyeong Baek, Kyungjae Andrew Yoon

**Affiliations:** 1 Department of Agricultural Biology, Seoul National University, Seoul 151-921, Korea; kongbob89@snu.ac.kr; 2 Research Institute of Agriculture and Life Sciences, Seoul National University, Seoul 151-921, Korea; 3 College of Pharmacy and Research Institute of Pharmaceutical Science, Gyeongsang National University, Jinju 660-701, Korea

**Keywords:** venom, solitary wasp, social wasp, peptide, protein, aculeata

## Abstract

The primary functions of venoms from solitary and social wasps are different. Whereas most solitary wasps sting their prey to paralyze and preserve it, without killing, as the provisions for their progeny, social wasps usually sting to defend their colonies from vertebrate predators. Such distinctive venom properties of solitary and social wasps suggest that the main venom components are likely to be different depending on the wasps’ sociality. The present paper reviews venom components and properties of the Aculeata hunting wasps, with a particular emphasis on the comparative aspects of venom compositions and properties between solitary and social wasps. Common components in both solitary and social wasp venoms include hyaluronidase, phospholipase A2, metalloendopeptidase, *etc*. Although it has been expected that more diverse bioactive components with the functions of prey inactivation and physiology manipulation are present in solitary wasps, available studies on venom compositions of solitary wasps are simply too scarce to generalize this notion. Nevertheless, some neurotoxic peptides (e.g., pompilidotoxin and dendrotoxin-like peptide) and proteins (e.g., insulin-like peptide binding protein) appear to be specific to solitary wasp venom. In contrast, several proteins, such as venom allergen 5 protein, venom acid phosphatase, and various phospholipases, appear to be relatively more specific to social wasp venom. Finally, putative functions of main venom components and their application are also discussed.

## 1. Introduction

Wasps present an extremely diverse group in the suborder Apocrita (Hymenoptera), which is conventionally divided into two groups: Parasitica and Aculeata [1,2]. The clade Parasitica comprises the majority of parasitoid wasps, whereas the clade Aculeata contains most parasitic and predatory wasps with their ovipositor completely modified into a stinger for injecting venom [1]. These stinging Aculeata wasps are further divided into two subgroups (solitary *vs.* social) depending their lifestyle in the context of sociality [3].

Approximately 95% of 15,000 species of Aculeata wasps are solitary and are widely distributed across various families in the Aculeata [4]. The lifestyle of solitary wasps is unsocial; they do not form colonies [4]. After mating, the female solitary wasp builds one or more nests, hunts preys, and stores them in the cell(s) of the nest as provisions for the young.

Most solitary wasps sting their prey to paralyze and preserve it to use as food for the hatched wasp larvae. Thus, the primary compositions of the solitary wasp venom are various bioactive molecules that have the functions of paralysis, antimicrobial activity, developmental arrest, *etc*. Although the term “solitary wasp” is not strictly interchangeable with “hunting wasp”, the solitary wasp in this review refers to the aculeate wasps that hunt prey for offspring but are not social in their lifestyle. As expected from the common lifestyle, the primary purpose of ectoparasitoid venom is similar to that of solitary hunting wasp. The ectoparasitoid wasp venom is also known to contain a variety of bioactive substances that cause paralysis/lethargy and developmental arrest of the host (reviewed in [5]).

Carnivorous social wasps represent only a small portion in the Aculeata. Social wasps form colonies and some species, such as hornets and yellow jackets, build very large nests. Unlike solitary wasps, social wasps usually sting to defend themselves and their colonies from vertebrate predators [6]. Once disturbed, the entire colony is mobilized via an attack pheromone to sting the intruder, resulting in mass envenomation, which can be fatal [7]. Most social wasps generally butcher their prey, mostly insects and spiders, without stinging it and bring the most nourishing parts of it back to the colony to feed larvae [8]. Therefore, social wasps do not need to paralyze and preserve hunted prey with their venom. Social wasp’s venom appears to have evolved to maximize the defense potential in the ways to intensify venom-induced pain and/or to augment allergenic and immune responses of humans or animals [6]. Since social wasp venom contains various molecules that cause hypersensitivity reactions, such as anaphylaxis, it has been of a great medical and clinical importance.

**Figure 1:**
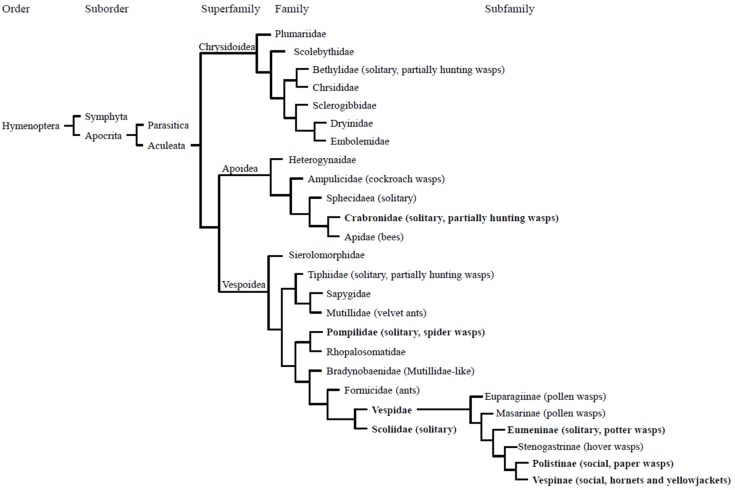
Composite cladogram showing taxonomic relationship of Aculeata [2,8,9]. Sociality (solitary or social) or common names are shown in parenthesis next to family of subfamily name. Non-labeled families are parasitoids. Bethylidae, Crabronidae, and Tiphiidae are mostly parasitoids, but some show hunting wasp-like behaviors (marked as partially hunting). Families or subfamilies reviewed in this article are highlighted with bold font.

The venoms of wasps have been considered as a potential source of novel bioactive substances for pharmacological, therapeutic, and agricultural uses [1]. Wasp venoms are known to contain three major groups of molecules, namely, (1) proteins, including enzymes and allergens; (2) small peptides with various functions, including neuro- and antimicrobial activities; and (3) low molecular weight substances, including bioactive amines, amino acids, *etc.* [1]. However, until now, key components that differentiate between the solitary and social wasp venom have not been accurately identified yet.

Since the venomic properties of parasitoid wasps (Pasasitica) have been well reviewed elsewhere [5], in the present review, we have focused on the venom of the Aculeata hunting wasps, particularly those in the superfamily Vespoidea (Figure 1), with a particular emphasis on the comparative aspects of venom proteins and peptides and their properties between solitary and social wasps.

## 2. Venom of Hunting Wasps

### 2.1. General Properties and Origin

Venom is a form of toxin secreted by animals that aims at the rapid immobilization or inactivation of their prey or enemy. Venom components target main critical systems of an organism, such as neuromuscular and hemostatic systems, to achieve the most efficient and rapid immobilization or death of the victim. Since venomous animals prey on many different species, as well as have a defense system against unspecified intruders, they produce various proteins and peptides both with specific molecular targets and those that are active across a wide range of animal species [10].

The remarkable similarities observed in the protein compositions between the venoms from various animal species strongly suggests that a variety of proteins have been commonly and convergently recruited in animal venoms throughout the evolution, and a protein suitable for recruitment has been under structural and/or functional constraints [11,12]. The recruitment and evolution of wasp venom components has satisfied the strict requirements of safety towards the venom-producing wasps and efficiency towards targets, which are largely dependent on individual, populational, and ecological factors [5]. Therefore, it is not surprising that there are many common components in the venoms of different wasp species.

Social wasp venoms (of which the most studied is the venom of the social Vespidae), in general, induce local edema and erythema caused by an increased permeability of the blood vessels in the skin, which is a net effect of active peptides, such as bradykinin-like peptides, mastoparans, and chemotactic peptides. These local reactions of venom peptides produce a prolonged pain that often continues for several hours and itching that can last for days. In addition to these direct effects of wasp stings, immunological reactions caused by venom allergens, such as: phospholipase A (A1 and A2), hyaluronidase; Cysteine-rich secretory proteins, Antigen 5 and Pathogenesis-related proteins (CAP); and serine proteases, have also been frequently observed. Allergic reactions lead to anaphylaxis and, by a large dose of venom, systemic toxic reactions, such as hemolysis, coagulopathy, rhabdomyolysis, acute renal failure, hepatotoxicity, aortic thrombosis, and cerebral infarction [13,14].

### 2.2. Solitary vs. Social Wasp Venom

The venoms of most solitary wasps are not lethal to the prey. Instead, the venoms induce paralysis and regulation of the prey development and metabolism to maintain the life of the prey while feeding wasp larvae [6]. By contrast, the main function of social wasp venoms appears to be that of defense. Venoms of both solitary and social wasps plays a defensive role by causing a number of symptoms in envenomed predators, including pain, tissue damage, and allergic reactions [6]. Pain is a most common defensive property of venoms, where social wasp venom produces a generally stronger pain than that of solitary wasps. In most cases, pain is accompanied by a potential tissue damage and impairment of normal body functions. In addition, venom contains a number of strong allergens, suggesting that venom allergenicity is likely to have a defensive value. Any wasp producing the venom that induces an acute and systematic allergic reaction (anaphylactic) in a predator can not only immediately stop the predator’s attack, but also traumatize that organism to avoid the wasp [6]. Based on the distinctive venom properties between solitary and social wasps, one can expect considerable differences in the main components from the point of venom function. Since many solitary wasps are specific in selecting prey species, it would be intriguing to investigate the differences in venom composition depending on prey species and the factors that influence the recruitment of different venom substances.

## 3. Comparative Studies of Solitary *vs.* Social Wasp Venoms

To characterize the venom of solitary and social hunting wasps, a comparative study of venom gland transcriptome and proteome is mandatory. However, most previous genomic or proteomic analysis has focused on parasitoid wasps. For example, among 44 Hymenoptera genome databases annotated at NCBI [15], only one is that of a hunting wasp: specifically, the European paper wasp, *Polistes canadensis*. Moreover, only a few studies have dealt with venom gland-specific transcriptomes or proteomes so far, and all of them are limited to the Vespidae family.

Herein, seven expressed sequence tag (EST) libraries and three proteome profiles and the putative functions of the Vespidae wasp venoms were compared and summarized: for solitary wasp venom, *Orancistrocerus drewseni* [16,17], *Eumenes pomiformis* [16,18], *Rhynchium brunneum* [19] (Table 1); for social wasp venom, *Vespa tropica* [19], *V. crabro* [20], *V. analis* [20], *V. velutina* [21], and *Polybia paulista* [22] (Table 2). In presenting putative functions of venom components in Table 1 and Table 2, biological functions in venom that have been experimentally tested are marked by underlines. The putative functions of remaining components have been implied on the basis of deduced amino acid sequence homology. Venom peptides of various social wasps in the Vespidae, which are reported individually without transcriptome or proteome analysis, are also reviewed in the next section (Table A1).

A total of 82 and 44 venom proteins (or genes) have been identified from solitary and social wasps, respectively (Table 1 and Table 2) and divided into several categories (e.g., Enzymes, Hemostasis affecting proteins, Muscle-related proteins, *etc.*). The lower number of venom proteins in solitary wasps does not necessarily indicate that solitary wasp venom components are less diverse that those of social wasps. Rather, this indicates that solitary wasp venoms may have been under-explored and the list in Table 1 is most likely less represented.

**Table 1:** Venom peptides and proteins from solitary wasps and their putative functions.

Protein/Peptide	Putative Function ^a^	Species ^b^	References
**Neurotoxins**			
α-pompilidiotoxin	Paralysis (Na^+^ channel blocking)	As	[23]
β-pompilidiotoxin	Paralysis (Na^+^ channel blocking)	Bm	[23]
Dendrotoxin-like	Paralysis (K^+^ channel blocking)	Ep	[16,18]
**Kinins**			
Wasp kinin	Pain production	Ca, Cd, Ci, Cm, Mf, Mp	[24,25,26]
**Mast cell degranulating peptides**			
Mastoparan-like	Allergic inflammation (Mast cell degranulation), Antimicrobial activity	Af, As, Ep, Er, Od1, Od2	[16,18,27,28,29,30,31,32]
**Chemotactic peptides**			
Wasp chemotactic peptide	Inflammatory activity Antimicrobial activity	Cf, Ep, Od1, Rb	[16,18,30,33]
**Enzymes**			
Acetyl-CoA synthase	Involvement in metabolism of acetate	Rb	[19]
Alcohol dehydrogenase	Oxidation of ethanol to acetaldehyde	Ep	[18]
Amidophosphoribosyltransferase	Regulation of cell growth	Rb	[19]
Arginine kinase	Paralysis	Cd, Od1, Ep, Rb	[16,19,34]
ATP synthase	ATP synthesis	Od1, Ep, Rb	[17,18]
Carboxylesterase	Lipid metabolism	Rb	[19]
Citrate synthase	Catalyzing the citric acid cycle	Rb	[19]
Cytochrome P450 monooxygenase	Metabolism of toxic compounds	Od1, Rb	[17,19]
DNA-directed RNA polymerase	Synthesis of mRNA precursor	Rb	[19]
Farnesoic acid *O*-methyltransferase	Regulation of biosynthetic pathway of juvenile hormone	Rb	[19]
Glutamate decarboxylase	Involvement in beta-cell-specific autoimmunity	Ep	[18]
Glyceraldehyde-3-phosphate dehydrogenase	Direct hemolytic factor	Rb	[19]
Glycogenin	Synthesis of glycogen	Rb	[19]
HECT E3 ubiquitin ligase	Regulation of cell trafficking	Ep	[18]
Hyaluronidase	Venom dissemination	Ep, Rb	[18,19]
Myo inositol monophosphatase	Regulation of inositol homeostasis	Rb	[19]
Phospholipase A2	Hydolysis of lecithins	Ep, Rb	[18,19]
Protein tyrosin phosphatase	Regulation of cellular processes	Rb	[19]
Serine/threonine protein phosphatase	Regulation of biochemical pathways	Rb	[19]
Tyrosine 3-monooxygenase	Regulation of dopamine synthesis	Od1, Ep	[16,18]
**Hemostasis affecting proteins**			
Metalloendopeptidase	Inhibition of platelet aggregation	Od1, Ep, Rb	[18,19]
Neprilysin	Inhibition of platelet aggregation	Rb	[19]
Serine protease/Chymotrypsin/Thrombin-like	Fibrinolytic activity Kinin releasing activity Melanization	Od1, Ep	[16]
**Muscle-related proteins**			
Actin	Regulation of hemocyte cytoskeleton gene expression	Od1, Ep, Rb	[16,19]
Ankyrin	Attachment of membrane proteins to membrane cytoskeleton	Rb	[19]
Bmkettin	Development of flight muscles	Od1, Rb	[17,19]
Calponin	Regulation of myogenesis	Od1, Ep, Rb	[17,18,19]
Muscle LIM protein	Regulation of myogenesis	Od1, Ep, Rb	[17,19]
Muscle protein 20 Myomesin	Regulation of muscle cotraciton	Od1, Ep	[17,18]
	Anchoring the thick filaments	Rb	[19]
Myosin heavy chain	Regulation of muscle functions	Od1, Ep, Rb	[17,18,19]
Myosin light chain	Modulation of the affinity of myosin for actin	Od1, Ep, Rb	[17,18,19]
Paramyosin	Regulation of thick filament in muscles	Od1, Ep, Rb	[17,18,19]
Titin	Assembly of contractile machinery in muscle cells	Od1, Rb	[17,19]
Tropomyosin	Muscle contraction	Od1, Rb	[17,19]
Troponin	Muscle contraction	Od1, Ep, Rb	[17,18,19]
Tubulin	Regulation of hemocyte skeleton genes expression	Od1, Ep, Rb	[17,19]
**Other proteins/peptides**			
Chemosensory protein	Transferring metabolism-related small molecules	Rb	[19]
Cytochrom C	Protein wire	Od1, Rb	[17,19]
Heat shock proteins	Prevention of protein misfolding	Od1, Ep, Rb	[16,19]
Insulin-like peptide binding protein	Developmental arrest (Inhibition of insulin signaling)	Ep	[18]
Sialin	Nitrate transporter	Rb	[19]
Sugar transporter	Maintenance of glucose homeostasis	Rb	[19]

^a^ The functional categories are either molecular, cellular or biological functions. When biological functions in venom are known, they were underlined; ^b^ Wasp species abbreviations: Af, *Anterhynchium flavomarginatum*; As, *Anoplius samariensis*; Bm, *Batozonellus maculifrons*; Ca, *Campsomeriella annulata*; Cd, *Cyphononyx dorsalis*; Cf, *Cyphononyx fulvognathus*; Ci, *Colpa interrupta*; Cm, *Carinoscolia melanosome*; Ep, *Eumenes pomiformis*; Er, *Eumenes rubronotatus*; Mf, *Megascolia flavifrons*; Mp, *Megacampsomeris prismatica*; Od1, *Orancistrocerus drewseni*; Od2, *Oreumenes decorates*; Pt, *Philanthus Triangulum*; Rb, *Rhynchium brunneum*.

**Table 2:** Venom peptides and proteins from social wasps and their putative functions.

Protein/Peptide	Putative Function ^a^	Species ^b^	References
**Neurotoxins**			
AvTx-7,8	Paralysis (K^+^ channel blocking)	Av	[35,36]
Agatoxin-like	Paralysis (Ca^2+^ channel blocking)	Vv1	[21]
Analgesic polypeptide	Paralysis (Na^+^ channel blocking)	Vv1	[21]
Calsyntenin	Paralysis (Ca^2+^ channel blocking)	Vc	[20]
Conophysin-R	Paralysis (Ca^2+^ channel blocking)	Vv1	[21]
Latrotoxin-like	Channel formation	Vv1	[21]
Leucine rich repeat domain-containing protein	Paralysis (Involvement in synaptic vescle trafficking)	Va2, Vc	[20]
Orientotoxin-like	Paralysis (Presynaptic effect, lysophospholipase activity)	Vo, Vv1	[21,37]
**Kinins**			
Wasp kinin	Pain production	Pa, Pc, Pe1, Pe2, Pf, Pi, Pj, Pm1, Pm2, Pp, Pr, Va2, Vc, Vm2, Vt, Vx	[20,25,38,39,40,41]
**Mast cell degranulating peptides**			
Mastoparan	Allergic inflamation (Mast cell degranulation)	Ap, Pe2, Pi, Pj, Pm2, Pp, Ps, Rs, Va1, Va2, Vb1, Vb2, Vc, Vd, Vl, Vm1, Vm2, Vo, Vt, Vv1, Vx	[19,20,38,42,43,44,45,46,47,48,49,50,51,52,53]
**Chemotactic peptides**			
Wasp chemotactic peptide	Inflammatory activity Antimicrobial activity	Ap, Pl, Pm2, Pp, Ps, Va2, Vb2, Vc, Vm1, Vm2, Vo, Vt, Vx	[20,38,40,41,45,49,50,52,54]
**Enzymes**			
Acetylcholinesterase	Pain processing (Hydrolysis of neurotransmitter)	Va2, Vc, Vv1	[20,21]
Acetyltransferase	Synthesis of acetylcholine	Vt	[19]
Acid phosphatase	Female reproduction	Va2, Vc	[20]
Acyl-CoA delta-9 desaturase	Insertion of double bond in fatty acids	Vt	[19]
AMP dependent coa ligase	Production of fatty acyl-CoA esters	Vt	[19]
Arginine kinase	Paralysis	Va2, Vc	[20]
Argininosuccinate synthase	Arginine synthesis	Vt	[19]
ATP-dependent protease	Mediation of protein quality	Vt	[19]
Carboxylesterase	Lipid metabolism	Va2, Vc	[20]
Chitinase	Chitinolysis	Va2, Vt	[19,20]
Core alpha 1,3-fructosyltransferase A	Glycoprotein production	Vt	[19]
Cytochrome P450 monooxygenase	Metabolism of toxic compounds	Vt	[20]
Dipeptidyl peptidase IV	Liberation of bioactive peptides	Va2, Vb1	[20,44]
Esterase FE4	Sequestration	Vc	[20]
Fatty acid synthase	Biosynthesis of hormones	Vt	[19]
Fibrinogenase brevinase	Fibrinolysis	Vv1	[21]
Glyceraldehyde-3-phosphate dehydrogenase Glycerol-3-phosphate acyltransferase Glycogenin GTP cyclohydrolase I isoform A	Direct hemolytic factor	Va2, Vc	[20]
	Synthesis of triacylglycerol	Vt	[19]
	Synthesis of glycogen	Va2, Vc	[20]
	Production of neurotransmitter	Vt	[19]
Hyaluronidase	Venom dissemination	Dm, Pa, Pp, Va2, Vc, Vm1, Vt, Vv3	[19,20,55,56,57]
Laccase	Oxidation, cuticle sclerotization	Vc	[20]
Myosin light chain kinase	Muscle contraction	Va2, Vc	[20]
O-linked n-acetylglucosamine transferase	Insulin signaling reduction	Vt	[19]
Peptidyl-prolyl cis-trans isomerase	Immune mediator	Vt	[19]
Phospholipase A1	Production of lipid mediator	Dm, Pa, Va1, Va2, Vc, Vv3	[20,57,58]
Phospholipase A2	Hydrolysis of lecithins	Va2, Vc, Vv1	[20,21]
Phospholipase B1	Hydrolysis of lysolecithins	Va2	[20]
Phospholipase D	Induction of inflammatory responses	Va2, Vc	[20]
Phospholipase DDHD	Synaptic organization	Va2, Vc	[20]
Purine nucleoside phosphorylase	Apoptosis of lymphocytes	Vt	[19]
Reverse transcriptase	Production of high venom yield	Vt	[19]
Thrombin-like enzyme	Coagulation factor	Va2, Vv1	[20,21]
γ-glutamyl transpeptidase	Apoptosis of ovariole cells	Va2, Vc	[20]
**CAP superfamily**			
Defensin	Antimicrobial activity	Va2, Vc	[20]
Venom allergen 5	Allergenic activity	Dm, Pa, Pe1, Pf, Va2, Vc, Vf, Vg, Vm1, Vm3, Vp, Vs, Vt, Vv2, Vv3	[20,56,57,59,60,61,62]
**Hemostasis affecting proteins**			
Blarina toxin	Production of kinins	Vv1	[21]
Coagulation factor Disintegrin	Platelet aggregation Platelet aggregation	Vv1 Va2	[21] [20]
Factor V activator	Coagulation factor	Vv1	[21]
Lectoxin-Enh4 Metalloendopeptidase Nematocyte expressed protein-6 Neprilysin	Anticoagulant factor Inhibition of platelet aggregation Inhibition of platelet aggregation Inhibition of platelet aggregation	Vv1 Va2, Vc, Vv1 Vv1 Va2, Vc	[21] [20,21] [21] [20]
Oscutarin-C	Fibrinolysis	Vv1	[21]
Ryncolin-3/4	Platelet aggregation	Vv1	[21]
Serine protease/Chymotrypsin/Thrombin-like	Fibrinolytic activity Kinin releasing activity Melanization	Va2, Vc, Vm1, Vt, Vv1	[19,20,21,63]
Snaclec	Platelet aggregation	Vv1	[21]
Vescular endothelial growth factor	Coagulation factor	Vv1	[21]
Veficolin	Platelet aggregation	Vv1	[21]
Venom plasminogen activator	Fibrinolysis	Vv1	[21]
Venom prothrombin activator	Fibrinolysis	Vv1	[21]
**Muscle-related proteins**			
Actin	Expression of hemocyte cytoskeleton	Va2, Vc	[20]
Calponin	Binding with actin	Va2, Vc	[20]
Muscle LIM protein	Regulation of myogenesis	Va2, Vc	[20]
Myosin heavy chain	Regulation of muscle functions	Va2, Vc	[20]
Paramyosin	Regulation of thick filament in muscle	Va2, Vc	[20]
Tropomyosin	Muscle contraction	Va2, Vc	[20]
Troponin	Muscle contraction	Va2, Vc	[20]
Vespin	Smooth muscle contraction	Vm1	[64]
**Protease inhibitor**			
Leukocyte elastase inhibitor isoform	Reduction of tissue damage	Vc	[20]
Serpin	Immune suppression (Inhibition of melanization)	Va2, Vc	[20]
**Other proteins/peptides**			
Anaphase-promoting complex subunit 13	Protein degradation	Vt	[19]
Apolipophorin-III	Lipid transport	Vt	[19]
Bhlh factor math 6	Regulation of developmental process	Vt	[19]
Bombolitin	Antimicrobial activity	Vc	[20]
CRAL/TRIO domain-containing protein	Regulation of cell growth	Vt	[19]
Cytochrome b	Transferring electrons	Vt	[19]
Doublesex isoform 1	Sex determination factor	Vt	[19]
Ejaculatory bulb-specific protein 3	Odorant binding protein	Va2, Vc	[20]
Elongation factor 2	Protein synthesis	Va2, Vc	[20]
Endopeptidase inhibitor	Inhibition of atrial natriuretic peptides	Vt	[21]
Endoplasmin	Protein folding	Va2, Vc	[20]
ETR-3 like factor 2	Pre-mRNA alternative splicing	Vt	[19]
Gigantoxin-1	Hemolytic activity	Vv1	[21]
Growth hormone inducible transmembrane protein	Apoptosis	Vt	[19]
GTPase-activating protein	Regulation of G protein signaling	Va2	[20]
Heat shock proteins	Prevention of protein misfolding	Vt	[19]
Insulin binding protein	Inhibition of insulin signaling	Va2, Vc	[20]
NADH-ubiquinone oxidoreductase chain 4	Involvement in respiratory chain	Vt	[19]
Natterin-4	Kininogenase activity	Vv1	[21]
Peptidoglycan-recognition protein 1	Antimicrobial activity	Vt	[19]
Phd finger protein	Protein-protein interaction	Vt	[19]
Plancitoxin	DNase activity	Vv1	[21]
Polyubiquitin	Proteolysis	Vt	[19]
SE-cephalotoxin	Paralysis	Vv1	[21]

^a^ The functional categories are either molecular, cellular or biological functions. When biological functions in venom are known, they were underlined; ^b^ Wasp spicies abbreviations: Ap, *Agelaia pallipes*; Av, *Agelaia vicina*; Dm, *Dolichovespula maculate*; Pa, *Polistes annularis*; Pc, *Polistes chinensis*; Pe1, *Polistes exclamans*; Pe2, *Protopolybia exigua*; Pf, *Polistes fuscatus*; Pi, *Parapolybia indica*; Pj, *Polistes jadwigae*; Pl, *Paravespula lewisii*; Pm1, *Paravespula maculifrons*; Pm2, *Polistes major*; Pp, *Polybia paulista*; Pr, *Polistes rothneyi*; Ps, *Protonectarina sylveirae*; Rs, *Ropalidia* sp.; Va1, *Vespa affinis*; Va2, *Vespa analis*; Vb1, *Vespa basalis*; Vb2, *Vespa bicolor*; Vc, *Vespa crabro*; Vd, *Vespa ducalis*; Vf, *Vespa flavopilosa*; Vg, *Vespula germanica*; Vl, *Vespula lewisii*; Vm1, *Vespa magnifica*; Vm2, *Vespa mandarina*; Vm3, *Vespula maculifrons*; Vo, *Vespa orientalis*; Vp, *Vespula pensylvanica*; Vs, *Vespula squamosa*; Vt, *Vespa tropica*; Vv1, *Vespa velutina*; Vv2, *Vespula vidua*; Vv3, *Vespula vulgaris*; Vx, *Vespa xanthoptera*.

### 3.1. High Throughput Identification of Wasp Venom Components

A recent introduction of the cost-effective RNA sequencing technology in conjunction with bioinformatics has enabled for a more extensive identification of genes encoding venom peptides and proteins from the transcriptome of venom gland, thus allowing for a more comprehensive understanding of venom composition. Initial high throughput analysis of venom gland transcriptome was conducted with parasitoid wasps (reviewed in [5]).

Recently, several papers have reported the transcriptomic analysis of venom gland of social wasps [20,21]. In the transcriptome of *V. velutina* venom, a variety of hemostasis-impairing toxins and neurotoxins were identified in addition to the genes encoding for well-known venom proteins and peptides. The most abundant hemostasis-impairing toxins consisted of two families based on their mode of action, namely: (1) the hemolytic toxins, including venom plasminogen activator, snaclec, lectoxin-Enh4, and fibrinogenase brevinase, *etc*.; and (2) the toxins involved in blood coagulation cascade, including factor V activator, oscutarin-C, ryncolin-3/4, veficolin, coagulation factor, thrombin-like enzyme, and venom prothrombin activator [21]. Second in abundance were neurotoxins in *V. velutina* venom, which were suggested to target either ion channels or synaptic components. Interestingly, genes encoding latorotoxin-orthologs were identified.

In a comparative analysis of the venom gland transcriptomes from two species of social wasps, *V. crabro* and *V. analis*, a total of 41 venom-specific genes were commonly identified in both transcriptomes; however, their transcriptional profiles were different [20]. These major venom components were identified and confirmed by mass spectroscopy. Most major venom genes, including prepromastoparan, vespid chemotactic precursor, vespakinin, *etc.*, were more predominantly expressed in *V. crabro*, whereas some minor venom genes, including muscle LIM protein, troponin, paramyosin, calponin, *etc.*, were more abundantly expressed in *V. analis*. Taken together, these results suggest that *V. crabro* venom is more enriched with major venom components and thus is potentially more toxic as compared with *V. analis* venom. Any gene encoding latrotoxin-like peptide, which was observed in the *V. velutina* venom transcriptome [21], was not annotated from the transcriptomes of *V. crabro* and *V. analis.* Some of the toxins involved in blood coagulation cascade, such as metalloendopeptidase and neprilysin, were identified in the venom gland transcriptomes of *V. crabro* and *V. analis.*

In the comparison of bioactivities of mastoparans, a family of major venom peptides, from these two hornets, *V. analis* mastoparan showed a significantly higher hemolytic activity, suggesting its higher cytotoxic potential as compared to *V. crabro* mastoparan [65]. In addition, *V. analis* mastoparan exhibited significantly more potent antimicrobial activities against *Escherichia coli* and *Candida albicans* and a significantly higher antitumor activity than *V. crabro* mastoparan. Such enhanced bioactivities of *V. analis* mastoparan are likely to be attributable to the additional Lys residue present in the mature peptide, as proposed by the secondary structure prediction [65]. Considering the potential differences in toxicity of each venom component, this finding further implies that, for a better understanding of venom toxicity, it would be necessary to investigate the structural properties of venom components and their quantitative profiles.

To the best of our knowledge, no large-scale transcriptomic analysis of venom gland from solitary wasp has been conducted so far. Instead, the suppression subtractive hybridization (SSH) technique has been employed to enrich the venom gene components of solitary hunting wasps, *O. drewseni*, *E. pomiformis*, and *R. brunneum* [17,18,19,66].

Hyaluronidase and phospholipase A2, which are known to be the main components of wasp venom, were found with high frequencies in the subtractive library of venom gland/sac of *O. drewseni* [17] along with other enzymes, including zinc-metallopeptidases. The latter are reported to induce hemolysis, hemorrhaging, and inflammation combined with phospholipase A2 and to contribute to prey immobilization and digestion [67,68]. Over 30% of contigs assembled from the library, including one with high abundance, had no BLAST hits, which appeared due to the lack of sufficient venom gland gene database and functional information on many of the venom components. Similarly, a subtractive cDNA library of the venom gland/sac of *E. pomiformis* was constructed by SSH [18]. Among 102 contigs assembled from the library, 37 contigs were annotated via BLASTx search and manual annotation, of which 8 contigs (337 ESTs) encoded short venom peptides (10 to 16 amino acids) occupying a majority of the library (62%) [18]. Genes encoding a novel dendrotoxin-like peptide containing the Kunitz/BPTI (bovine pancreatic trypsin inhibitor) domain, which is known to be K^+^ channel blockers, were identified from the library of *E. pomiformis* venom gland (named EpDTX). In addition to the major components of wasp venom (*i.e.*, hyaluronidase and phospholipase A2), several transcripts encoding metallopeptidases and decarboxylase, which are likely to be involved in the processing and activation of venomous proteins, peptides, amines, and neurotransmitters, were identified [18]. Interestingly, a transcript encoding a putative insulin/insulin-like peptide binding protein (IBP), which was suggested to be involved in growth regulation of their prey, was identified as well.

A proteomic approach in conjunction with the transcriptome analysis has been attempted for the identification of venom components in *E. pomiformis* and *O. drewseni.* The venom proteins were identified by one-dimensional sodium dodecyl sulfate polyacrylamide gel electrophoresis (SDS-PAGE), which was followed by nano electrospray ionization-tandem mass spectrometry (ESI-MS/MS) for protein identification. Over 30 protein bands (2–300 kDa) were detected from the crude venom of each wasp [16]. With the aid of the venom gland/sac-specific cDNA database, a total of 31 and 20 proteins were identified from *E. pomiformis* and *O. drewseni* venoms, respectively. The arginine kinase, in both full-length and truncated form, was predominantly found in both wasps’ venoms. However, IBP was found in abundance only in *E. pomiformis* venom, reflecting its unique behavior of oviposition and provision, which is different from the subsocial *O. drewseni* [69].

Genes differentially expressed in the venom of social (*V. tropica*) and solitary (*R. brunneum*) wasps were investigated by a comparative transcriptome analysis based on SSH [19]. Although no remarkable differences in the distribution of functional categories were observed between the two venom gland/sac cDNA libraries, some groups of genes were found specific to either *V. tropica* or *R. brunneum*. When summarized together with the other transcriptomic and proteomic venom studies overviewed above and the proteome profiles of *P. paulista* [22], it can be concluded that venom allergen 5 and acid phosphatase are specifically present in the social wasp venom, whereas several venom peptides, IBP, and dendrotoxin are more specifically found in the solitary wasp venom gland (Table 1 and Table 2).

To summarize, the combined approach of the venom gland transcriptome analysis, either by full-scale RNA sequencing or SSH-based enriched library sequencing, and the direct analysis of venom components by electrophoresis in conjunction with mass spectrometry has been most widely used to identify venom components, particularly, minor components, of both solitary and social wasps. Annotation of the venom gland/sac cDNA library in conjunction with *de novo* sequencing of venom peptides followed by transcript structure analysis is a useful approach for confirming the structure of a mature venom peptide and for identifying various novel venom peptides, including those rarely detected by proteomic analysis [66]. While an increasing body of genomic or transcriptomic information on wasp venom toxins has become available to date through the Hymenoptera Genome Database [70], the information on solitary wasp venom is relatively more limited as compared with that on social wasp. This situation highlights the importance of establishing the venom gland transcriptome database of solitary wasps, which is also a prerequisite for the proteomic/peptidomic identification of venom components. The availability of high-throughput technologies for the identification of venom constituents would also facilitate the understanding of the evolution of solitary wasp-prey interactions. However, it should be noted that, for the comparative analysis of venom compositions on the basis of venom gland transcriptome or venom proteome/peptidome, the same criteria should be employed for all analytical procedures (*i.e.*, sample collection, preparation and depth of sequencing or mass spectrometry) to avoid any bias due to the differences in sample and data quality. A guideline for sample preparation can be suggested based on previous studies. In case of venom transcriptome analysis, to obtain 1–10 μg of high quality total RNA for conventional RNA sequencing, dissection of 1–20 venom glands, depending on the size of wasp, from live wasps is recommended [17,18,19,20,21,71]. For venom proteome/peptidome analysis, a sufficient amount of venom can be extracted from 2 to 10 isolated venom sacs (depending on the size of wasp) by centrifugation (1000–10,000 × *g* for 5~10 min) [16,18,20,72].

### 3.2. Comparative Aspects of Venom Components between Solitary vs. Social Wasp

Contents of venom components can be directly estimated from their proportion in the proteomic/peptidomic profiles of venom. Alternatively, they can also be indirectly predicted from the proportions of their transcripts in the transcriptome or cDNA library of venom gland. Previous comparisons of the relative contents of venom components suggest that venom peptides (mastoparan-like and chemotactic peptide-like), metalloendopeptidase, hyaluronidase, and arginine kinase are predominantly found in solitary wasps [16,18,19] while mastoparan, vespid chemotactic peptide (VCP), venom allergen 5, serine protease, and hyaluronidase were generally ranked as top five components in social wasps [19,20].

Both solitary and social wasp venoms contain several common components, including hyaluronidase. Hyaluronidase is hydrolase that can hydrolyze the viscous hyaluronic acid polymer, an important constituent of the extracellular matrix of all vertebrates [73]. Hyaluronidase is also commonly found in the venoms of various venomous organisms, including bees and wasps. Due to the degradation of hyaluronic acid in the extracellular matrix, venom hyaluronidase functions as a diffusion factor that facilitates the diffusion of injected venom from the site of sting into circulation, thereby potentiating the action of other venom ingredients [74,75]. Besides, hydrolyzed hyaluronan fragments are known to stimulate inflammation, angiogenesis, and immune response, thereby resulting in a quicker systemic envenomation [76]. Owing to its role in venom spreading, hyaluronidase is common not only in social wasp venoms, but also in solitary wasp venoms as an essential venom ingredient (Table 1 and Table 2). In addition to hyaluronidase, other venom proteins that are commonly found in both wasps include phospholipase A2, metalloendopeptidase, *etc.*, which are also well-known allergens.

#### 3.2.1. Solitary Wasp Venom-Specific Features

Many peptides, which constitute a sizeable portion in solitary wasp venom, are not exactly categorized in the well-defined groups of social wasp venom peptides, such as kinin, mastoparan, or chemotactic peptides. Further detail on individual venom peptides is provided in Section 4. Although it has been expected that more diverse bioactive components with the functions of prey inactivation and physiology manipulation are present in solitary wasps, available studies on venom compositions of solitary wasps are simply too scarce to generalize this notion. Nevertheless, several neurotoxic peptides or proteins appear to be specific to solitary wasps and are involved in prey paralysis.

Non-kinin neurotoxic peptides isolated from Pompilidae solitary wasps [α-pompilidotoxin (α-PMTX) from *Anoplius samariensis* and β-PMTX from *Batozonellus maculifrons*] [23,77] are known to affect both vertebrate and invertebrate nervous systems by slowing or blocking sodium channel inactivation [78], thereby paralyzing cockroach prey.

A novel dendrotoxin-like peptide containing the Kunitz/BPTI domain was identified from in *A. samariensis* (As-fr-19), *E. pomiformis* (EpDTX), and *R. brunneum* venoms [16,18,19,79]. Since dendrotoxin is known to block K^+^ channel, the presence of dendrotoxin-like venom peptides in solitary wasp venoms suggests its involvement in the paralysis of prey.

More extensive research on the components of solitary wasp venom is likely to identify other neuroactive peptides with similar functions. However, it is also worth mentioning that low molecular mass substances functioning as neurotoxin or neuromodulator, such as philanthotoxins found in the Egyptian solitary wasp *Philanthus Triangulum* venom [80,81,82] and biogenic amines, [e.g., γ-aminobutyric acid (GABA), taurine, and β-alanine] found in the jewel wasp *Ampulex compressa* venom [83], can play a more decisive role than neuroactive venom peptides in solitary wasps.

In particular, IBP was found to be a major component (22.4% of total venom protein) in the venom of a solitary wasp *E. pomiformi* [16]. To elucidate biological and molecular functions of EpIBP, EpIBP and its homologous protein of *Spodoptera exigua* (SeIBP) were *in vitro* expressed using an *E. coli* expression system [84]. *S. exigua* larvae injected with EpIBP exhibited an increased survivorship and a reduced loss of body weight under a starvation condition. Both EpIBP and SeIBP were found to interact with apolipophorin III (apoLp III), implying that EpIBP might control the apoLp III-mediated metabolism, thereby regulating the growth of prey [84]. Similarly, an IBP was also identified in the venom of a parasitoid wasp *Nasonia vitripennis* and suggested to inhibit the growth of the host [85]. Although IBP was found in the venom gland transcriptomes of *V. crabro* and *V. analis*, its transcription level, as judged by the FPKM value, was very low [20]. Considering that the expression level of IBP in a solitary wasp *E. pomiformi* is much higher [16], IBP in social wasp venom with a negligible level of expression is likely to be non-functional.

Vitellogenin-like protein, which is known to be involved in immune stimulation by enhancing melanin synthesis [86], was identified from both *E. pomiformis* and *O. drewseni* venoms, suggesting that they are likely involved in the protection of prey from microbial invasion [16].

#### 3.2.2. Social Wasp Venom-Specific Features

Venom allergen 5 proteins, venom acid phosphatase, and various phospholipases (A1, B1, D, and DDHD) appear to be relatively more specific to social wasp venom (see Table 2). Together with hyaluronidase and acid phosphatase, venom allergen 5 protein is one of the major allergenic Vespid venom proteins [87]. It is reported that venom allergen 5 is a major venom component in social wasps and has been identified in all social wasps examined in the present review (see Table A1). Although the gene encoding venom allergen 5 is found from a solitary wasp *R. brunneum* [88], it was not identified at all in the venom gland/sac transcriptome and proteome libraries of solitary wasps, including *R. brunneum* [16,17,18,19], suggesting that it is not common in solitary wasps. Phylogenetic analysis of venom allergen 5 proteins of several Hymenopterans suggests that the gene can date back to the common ancestor of the Ichneumonoidea and the Aculeata, indicating its ancient origin [88]. Since all of the Vespid venom allergen 5 proteins show >57% of sequence identity, the tertiary conformations and allergenic capacities may not significantly differ [20,59]. Thus, a high degree of cross-reactivity in serological testing was observed among the venom allergen 5 proteins of the common group of yellow jackets and among those of the two common North American subgenera of paper wasps [59].

Venom acid phosphatase belongs to the enzyme group which hydrolyzes phosphomonoesters at acidic pH. It has been characterized as a glycoprotein causing histamine release from sensitized human basophils, as well as an acute swelling and flare reaction after intradermal injection into the skin of allergenic patients [89,90,91,92]. Venom acid phosphatase is a high-molecular-weight protein composed of 404–411 amino acid residues in the order Hymenoptera [91,93,94]. Venom acid phosphatase is not common in solitary wasp venoms (Table 1), whereas its gene was found in the venom gland transcriptomes of *V. crabro* and *V. analis* (Table 2), suggesting its relatively wider distribution in social wasp venoms and its importance as a major allergen.

Acetylcholine, a neurotransmitter and neuromodulator, is commonly found in social wasp venom and is likely to be involved in pain processing [95]. Acetylcholinesterase, which hydrolyzes the acetylcholine, was specifically identified in social wasps [96], suggesting that it may play a role in the regulation of pain sensation in envenomed vertebrates.

A number of venom proteins tentatively associated with hemostasis have been identified by the venom gland transcriptome analysis of *Vespa* social wasps (Table 2, [20,21]). A relatively fewer numbers of hemostasis-related proteins have been identified from solitary wasps (Table 1), suggesting that this group of proteins are likely to be associated with social defense by disrupting hemostasis of vertebrate predators.

## 4. Venom Peptides

Among a variety of venom components, peptides with a molecular weight range of 1.4–7 kDa are predominant in wasp venoms, comprising up to 70% of the dried venoms [14,18,19,20,66]. In addition to neurotoxic peptides including wasp kinins, a series of amphipathic α-helical peptides (mastoparans and chemotactic peptides) are also major peptidergic components. These venom peptides commonly exist in both solitary and social wasp venoms. However, no kinins have been identified yet in Vespidae solitary wasps. Only three chemotactic peptide-like peptides have been reported in Vespidae solitary wasps so far, implying that kinin-like peptides are not likely a major component in Vespidae solitary wasp venoms (Table A1).

Kinins, mastoparans, and chemotactic peptides share a common secondary structure: an *N*-terminal signal sequence, a prosequence, a mature peptide, and/or an appendix G or GKK at the *C*-terminus. They are post-translationally processed via the sequential liberation of dipeptides (A/P-X-A/P-X) in the prosequence by dipeptidyl peptidase IV (DPP-IV) [44,97]. Additionally, some of mastoparans and chemotactic peptides are further processed at C-terminal G or GKK residues via the *C*-terminal amidation catalyzed by peptidylglycine α-hydroxylating monooxygenase and peptidyl-α-hydroxyglycine α-amidating lysase [98,99]. Although many venom peptides pass through a similar posttranslational processing, the matured peptides have distinguishable structures and bioactivities. Moreover, several venom peptides matured via the same processing are not even categorized into kinin, mastoparan, or chemotactic peptides in solitary wasps (e.g., 6 venom peptides from *O. drewseni* and *E. pomiformis* in “Uncategorized Peptides” in Table A1). Since peptides are major components of both solitary and social wasp venoms, their kinds, properties, and putative functions are reviewed in detail in this section.

### 4.1. Neurotoxic Peptides

Neurotoxic peptides modulating ion channel and receptor functions have been described in wasp venoms. The first neurotoxin component that was isolated from wasp venom was a nicotinic acetylcholine receptor (nAChR) inhibitor, kinin. In 1954, the first wasp kinin was isolated from a social wasp, *Vespa vulgaris* [100]. Afterwards, many kinins of the Vespoidea wasp venoms were found to be responsible for the pain and paralysis after a wasp sting [24,101]. Until now, most of the neurotoxic peptides of hunting wasps are kinins. Wasp kinins will be discussed further in Section 4.2.

There are 2 non-kinin neurotoxic peptides isolated from Pompilidae solitary wasps: α-PMTX from *A. samariensis* and β-PMTX from *B. maculifrons* [23,77]. PMTXs, 13-amino acid venom peptides, affect both vertebrate and invertebrate nervous systems by slowing or blocking sodium channel inactivation [78]. α-PMTX greatly potentiates synaptic transmission of lobster leg neuromuscular junction by acting primarily on the presynaptic membrane [77]. Interestingly, β-PMTX, in which the lysine at 12 position of α-PMTX was replaced with arginine, is 5 times more potent than α-PMTX [23].

Recently, novel venom peptides, AvTx-7 and AvTx-8, were also reported as neurotoxins of the social wasp *Agelaia vicina* [35,36]. Although their primary structures have not been elucidated so far, they seem to be new types of venom peptides, different from kinins as judged by their distinct neural activity. AvTx-7 stimulates glutamate release through K^+^ channel and AvTx-8 inhibits GABAergic neurotransmission, whereas wasp kinins block nAChR.

### 4.2. Kinins

Bradykinin was first reported in 1949 as a mammalian blood serum substance that triggers a slow contraction of the guinea-pig ileum [102]. This nonapeptide acts on smooth muscles with contractions or relaxations. In the neuronal cells of vertebrates, bradykinin evokes a release of neuropeptides (galanin, neuropeptide Y, and vasoactive intestinal peptide) and catecholamines (dopamine, norepinephrine, and epinephrine) by depolarizing nerve terminals [25,103].

Bradykinin-like peptide was found in wasp venoms as the first neurotoxic and pain-producing peptide [100]. It irreversibly blocks the synaptic transmission of nAChR in the insect central nervous system (CNS) [104,105]. While wasp kinins have remarkable sequence similarities to the main structure of mammalian bradykinin [-PPGF(T/S)P(F/L)-], most of them are longer than bradykinin or differ at position 3 or 6, where proline is replaced by hydroxyproline or serine is replaced by threonine (Thr^6^-bradykinin), which has a single extra hydroxyl or methyl group, respectively. By the single amino acid substitution, Thr^6^-bradykinin displayed 3-fold higher anti-nociceptive effects on the rat CNS and remained active longer than bradykinin [106]. Considering their action on nAChR, bradykinins in solitary wasp venoms may play a crucial role in paralyzing prey during hunting [24].

Almost all social wasp venoms may have kinin or kinin-like activities, while, among solitary wasps, *Cyphononyx dorsalis* (Pompilidae) and several Scoliidae wasps have Thr^6^-bradykinin in their venom, and bradykinin was found only in the *Megacampsomeris prismatica* (Scoliidae) venom [24,107]. Since one of major pharmacological effects of kinins is pain sensation in vertebrates [108], the ubiquitous distribution of kinins in social wasp venoms suggests that kinins may function as a major defense and alarm device by generating pain in the envenomed vertebrate predators. The presence of kinins in the venom of Vespidae and Scoliidae, as well as that of Formicidae (ants), and no kinin-like activities in bees (Apidae) and bee-related solitary wasps (Crabronidae and Sphecidae) was supposed to support the suggestion that these three families are associated by synapomorphies [2,25]. Later on, however, kinin-like activity was also found in Ampulicidae (*A. compressa*) that is closely located to Apidae [109], and no kinins have been isolated from solitary wasps in Vespidae so far. Thus, the relationship between the venom kinins and evolution remains obscure, requiring a more extensive identification and characterization of Hymenopteran venom kinins.

### 4.3. Mastoparans

The most abundant peptide component of hunting wasp venoms, both in solitary and social wasps, is mastoparan. Of note, however, mastoparans have been thus far isolated only in Vespidae (both social and solitary), not in other solitary hunting wasp families. Mastoparans (mostly tetradecapeptides) act on mast cells to liberate granules and release histamine (mast cell degranulation, MCD), resulting in inflammatory response [110]. Their structural properties, net positive charge, and amphipathic α-helical structure, in which all side chains of the hydrophobic amino acids are located on one side of the axis, and those of the basic or the hydrophilic amino acid residues are on the opposite side, allow them to attach to biomembranes and form pores via barrel-stave, carpet, or toroidal-pore mechanisms, resulting in an increase of cell membrane permeability [111]. Mastoparans are often highly active against the cell membranes of bacteria, fungi, and erythrocytes, as well as mast cells, resulting in antimicrobial, hemolytic, and MCD activities. Meanwhile, MCD by mastoparan may occur also through the exocytosis of granules, triggered by mastoparan modulating G-protein activity without receptor interaction [112]. The net effect of MCD depends on the cell types: the secretion of histamine from mast cells, serotonin from the platelets, catecholamines from chromaffin cells, prolactin from the anterior pituitary, and even insulin from pancreatic β-cells [51,110,113]. Cell lytic activity also varies depending on the cell types. Generally, antimicrobial activity of mastoparans is higher against fungi than Gram-negative bacteria, *E. coli* [45,114]. In addition, probably due to the cell lytic activity against insect cells, antimicrobial mastoparans also caused feeding disorder in caterpillars, although they are not active against human erythrocytes [114].

Cell lytic activity of mastoparans also leads to mitochondrial permeability transition that affects cell viability and triggers tumor cell cytotoxicity (reviewed in [115]). Besides MCD and cell lytic activity, mastoparans also stimulate phospholipases A, C and D, mobilization of Ca^2+^ from mitochondria and sarcoplasmic reticulum, and necrosis and/or apoptosis [115,116]. A variety of biological functions of mastoparans have attracted attention to them as components for potential therapeutic and biotechnological applications in biomedicine (reviewed in [115,117]). Due to the lack of cell specificity, however, mastoparans could not be used as they are. That is, they would damage not only tumor cells, but would also negatively affect healthy cells. Accordingly, researchers are developing a delivery system for venom peptides targeting tumor cells and a selective release system inside tumor cells that would make venom peptides accumulate in a specific and controlled manner [118].

### 4.4. Chemotactic Peptides

The second major peptide group in hunting wasp venom is chemotactic peptides. Similarly to mastoparans, venom chemotactic peptides have also been isolated only from social and solitary wasps in Vespidae, not from other solitary wasp families. Like mastoparans, venom chemotactic peptides are generally tridecapeptides with an amphipathic, α-helical, linear, cationic, and *C*-terminal amidated secondary structure. Their primary activity is described as inducing cellular chemotactic response in polymorphonuclear leukocytes and macrophages [119] and, due to the structural homology, chemotactic peptides often reveal mastoparan-like MCD, antimicrobial, and hemolytic activities. Chemotactic activity results in a mild edema, accompanied by an inflammatory exudate around the stinging site, where polymorphonuclear leukocytes are mainly concentrated. In other words, chemotactic peptides do not directly trigger pain, but enhance the inflammatory response by wasp stings [1]; therefore, they are likely to be involved in defense. Their widespread distribution in most social wasp venoms supports this prediction.

Although chemotactic peptides are a major venom component, only three of them have been reported in the venom of solitary hunting wasps: Orancis-protonectin (OdVP2) [30,66], EpVP6 [18], and the one found in *R. brunneum* (named RbVP1 hereafter) [19]. These solitary wasp venom peptides were categorized into chemotactic peptides based on the amino acid sequence homology with the previously known peptides, without a chemotaxis analysis. Thus, they indeed should be further evaluated to be referred to chemotactic peptides.

There is no known conserved main structure for recognition of venom chemotactic peptides. In Table A1, venom chemotactic peptides reported in solitary and social hunting wasps are summarized and compared, revealing representative motives XX(G/R)XX, XX(G/A/S/R/K/T)(G/T/K/S)XX or, sometimes, an overlapped form of the two motives, where X is a hydrophobic amino acid, most frequently, Ile or Leu. While only some mastoparanshave these motives, all chemotactic peptides have them. In addition, chemotactic peptides possess no or only one Lys residue, rarely 2 (RbVP1 and HR2), while most of mastoparans have 2 or 3 Lys residues. Mastoparans with 3 Lys residues generally have a single Lys (-K-) and separately double Lys residues (-KK-). These characteristics were inferred from the sequences collected during the preparation of the present manuscript, thereby the two motives suggested above are not fully confirmed yet.

### 4.5. Other Venom Peptides

Many peptides in solitary wasp venom are not exactly categorized in kinin, mastoparan, or chemotactic peptides. Most of them are not functionally analyzed.

Amphipathic linear cationic α-helical peptides anoplin and decoralin, found in a Pompilidae wasp *A. samariensis* and an Eumeninae wasp *Oreumenes decoratus*, respectively, commonly have MCD and antimicrobial activities [27,28,120]. Anoplin has hemolytic activity as well [121].

Non-helical coil venom peptides OdVP4, EpVP3, EpVP3S, EpVP4a, EpVP4b, and EpVP5 of Eumeninae wasps have neither antimicrobial and hemolytic, nor insect cell lytic activities [18,66,114], which implies that those peptides might have novel properties other than cell lytic activity.

Bioactivities of As-peptide126, Cd-125 and Cd-146 [107], isolated from Pompilidae wasps, have not been evaluated so far. Another *A. samariensis* venom peptide As-fr-19, as well as its homologue EpDTX of *E. pomiformis*, has a sequence similarity to potassium or calcium channel blocker, dendrotoxins from snakes, cone snails, and sea anemones [18,79]. The precise biochemical functions of As-fr-19 and EpDTX have not been clarified so far, but they are likely to function as neurotoxins.

Vespin of *Vespa magnifica*, a 44 amino-acid peptide, exerts contractile effects on isolated guinea pig ileum smooth muscle by interacting with bradykinin receptors [64]. However, vespin does not share the conserved motif of kinins [-PPGF(T/S)P(F/L)-], suggesting that vespin is a novel kind of venom peptide with kinin-like activity.

Recently, genes encoding putative neurotoxic peptides (*i.e.*, agatoxin-like, conophysin-R-like, latrotoxin-like and orientotoxin-like) have been identified from the venom transcriptome of *V. velutina*, though their transcription levels were very low [21]. Neurotoxic effects of these tentative venom peptides remain to be addressed in further research.

## 5. Useful Wasp Venom Components for Pharmacological, Medical, and Agricultural Applications

Considering the huge diversity of wasp venom components, wasp venoms can be employed as a rich source of novel bioactive substances for pharmacological, therapeutic, and agricultural applications [1]. Some venom proteins and peptides have been exploited as candidates for the discovery of novel therapeutic agents. Furthermore, studies on social wasp venoms have provided crucial information on the main allergenic molecules that are responsible for the hypersensitivity reaction in humans and enabled for the development of immunotherapy for preventing venom-induced anaphylaxis [122,123]. Similarly to the venom of parasitoid wasps, venoms of solitary hunting wasps are also known to contain various substances that can manipulate the physiology of prey [16,84,124]. Such regulatory molecules produced by wasps would serve as innovative leads for developing novel, environmentally safe insect control agents [124].

### 5.1. Antimicrobial Agents

Antimicrobial peptides (AMPs), which are relatively small (<10 kDa), cationic, and amphipathic peptides, are a basic humoral immune component of most organisms, including wasps, against invading microbial pathogens [125,126]. These AMPs exhibit a broad-spectrum antimicrobial activity against various microorganisms, including Gram-positive and Gram-negative bacteria, protozoa, yeast, and fungi [127]. Over the last several decades, a number of AMPs, mostly belonging to the groups of mastoparans, VCPs, and kinins, have been isolated from a wide variety of wasp species [50]. Most AMPs with the origin of wasp venom belong to the peptides forming alpha-helical structures, or coils rich in cysteine residues [111,114] and are suggested to act by perforating the plasma membrane, thus resulting in the cell lysis and death [111].

Mastoparan or mastoparan-like peptides are the alpha-helical peptides and have been identified in a wide range of wasps, including both solitary and social wasps [16,18,19,20,27,28,29,30,31,32,38,42,43,44,45,46,47,48,49,50,51,52,53]. The AMP from the Brazilian wasp *P. paulista* venom (MP1) has a broad-spectrum antibiotic activity against Gram-negative and Gram-positive bacteria without showing apparent hemolytic and cytotoxic activities [42]. The applicability of mastoparans for therapeutic and biotechnological use has been also reviewed elsewhere [115].

Three venom peptides (OdVP1, OdVP2 and OdVP3) isolated from the venom of the solitary wasp *O. drewseni* showed the typical features of amidated C-termini proteins and had a high content of hydrophobic and positively charged amino acids, resembling the amphipathic α-helical secondary structure of mastoparans [66]. Despite the distinctive sequences context in mature peptide, the overall transcript structure of the OdVPs showed a high similarity to that of *Vespa basalis* mastoparan-B by containing a signal sequence, a prosequence, a mature peptide, and a *C*-terminal glycine [66]. The OdVPs exhibited strong activities against fungi, but weak antibacterial activities. OdVP2L, having additional Glu–Pro residues, showed a high antifungal activity against the gray mold *Botrytis cinerea*, but did not show antimicrobial activity against bacteria or Gram-positive yeast [66]. Venom peptides of a-helical structure from a solitary wasp *E. pomiformis* (EpVP1, EpVP2a, EpVP2b, and EpVP6) also exhibited varying degrees of anti-microbial activities against Gram-negative *E. coli*, Gram-positive *Staphylococcus aureus*, Gram-positive yeast *C. albicans*, and the gray mold *B. cinerea* [114].

Since microbial infection mediated by biofilms has been a major problem in the use of implantable devices, several approaches, including the covalent immobilization of AMPs, have been attempted to tackle this problem [128]. To this end, the immobilization of MP1, a broad-spectrum AMP from a social wasp *P. paulista* venom, onto silicon surfaces has been attempted via the “allyl glycidyl ether brush”-based polymerization chemistry [129]. The antibacterial activity of the MP1-immobilized surfaces was retained after 3 days of incubation in artificial urine without causing any significant cytotoxicity against human red blood cells, suggesting the stability and safety of the AMP coating in physiological environments [129]. Based on this finding, a general approach to exploit and immobilize other AMPs as novel surface-sterilizing agents can be attempted.

### 5.2. Antitumor Agents

Mitoparan, a synthetic mastoparan analog, can form pores in the cancer cell plasma membrane and eventually lead to its death either by necrosis or by triggering apoptosis [130,131]. Due to their non-specific cytolytic activity and instability when injected in blood, however, the use of cytosolic peptides, such as mitoparan, is limited [118]. To overcome this limitation, Moreno *et al.*, (2014) have devised a pro-cytotoxic system based on mitoparan conjugated to poly(l-glutamic acid) PGA polymer through specific cleavage sequences that are cleaved by overexpressed tumor proteases, such as the metalloproteinase-2 or cathepsin B, in which the conjugated mitoparan becomes active only when it reaches cancer cells, then is cleaved and released by the tumor proteases [118].

The MP1 AMP, a mastoparan-like pore-forming peptide, has been determined to have highly selective antitumor activities against several types of cancer cells, including bladder and prostate cancer cells [132] and multidrug-resistant leukemic cells [133]. Recently, the highly specific antitumor activity of MP1 was determined to be due to its selective affinity to phosphatidylserine (PS) and phosphatidylethanolamine (PE), thereby enhancing the MP1-driven poration of cancer cell membrane, in which the outer lipid bilayer has an enriched PS and PE composition [134]. When combined with other anticancer drugs, the selectivity of MP1 peptide to disturb the cancer cell membrane may provide synergistic potentials, which can dramatically improve the therapeutic efficacy [134]. The mastoparans from social wasps *V. crabro* and *V. analis* also exhibited antitumor activities against ovarian tumor cells, with *V. analis* mastoparan showing a greater antitumor activity [65]. Taken together, mastoparan-like peptides from wasps can serve as good candidates for lead compounds of novel anticancer drugs [134].

### 5.3. Venom Allergy Diagnosis and Immunotherapy

Hymenoptera venom allergy (HVA) is an anaphylactic reaction of human to stings of social Hymenopteran insects, including honey bees, yellow jackets, hornets, bumble bees, and paper wasps [122]. A small but significant portion (0.3%–3.4%) of general human population is known to show systemic allergic reactions to Hymenoptera stings [122]. While whole venoms have been usually used for both diagnosis and immunotherapy, their diagnostic precision, when based on whole venom preparations, has been often impaired by immunoglobulin E (IgE) cross-reactivity between different venoms, which might be due to highly conserved venom allergens present in venom of different families or due to the presence of common cross-reactive carbohydrate determinants on venom allergens [122,135]. Nevertheless, the information on single venom allergens for diagnostic and therapeutic purposes has been limited, impeding an in-depth understanding of molecular basis underlying HVA. Recent employment of omics technologies for venom study has enabled a rapid discovery of novel venom components of medical importance and, thus allowed for a better molecular understanding of the entire “venome” as a system of unique and characteristic components [122]. Recombinant allergens, such as phospholipase A1, hyaluronidase, and venom allergen 5, have been generated from four important genera in Vespidae (*i.e.*, Vespula, Dolichovespula, Vespa, and Polistes) and used for diagnosis (reviewed in [136]). When the IgE-binding capacity of recombinant and purified natural venom allergens was compared, recombinant allergens exhibited higher specific responses without cross-reactivity and false positive results, indicating that they are better than highly purified natural preparations in terms of the clinical relevance of an individual allergen [73,137]. The potential of recombinant allergens for diagnostic and therapeutic applications has been well reviewed by Muller (2002) [136]. The use of cocktails with recombinant allergens for diagnosis can significantly increase the specificity of conventional diagnostic tests, such as immediate-type skin tests and the assays for serum-specific IgE antibodies [136]. The hypoallergenic mutants or modified variants of major venom allergens or the T-cell epitope peptides generated by recombinant technologies can be used as vaccines for immunotherapy to treat HVA [57,136].

### 5.4. Biopesticides

Manipulation of host by parasitoid wasp venom can be achieved via a variety of means, such as transient paralysis, immune suppression, endocrine dysfunction, metabolic alteration, and developmental arrest (reviewed in [124]). Several peptide/protein neurotoxins, including AvTx, pompilidotoxin, agatoxin-like, latrotoxin-like, orientotoxin-like, dendrotoxin-like peptides, among others, have been identified in solitary and social wasp venoms, of which those from solitary wasps (*i.e.*, pompilidotoxin and dendrotoxin-like peptide) are known to be involved in prey paralysis (Table 1 and Table 2; [16,18,23,77,78,138,139]). However, only one venom component that can regulate prey physiology has been identified and characterized in solitary wasps [16,18,51,84]. Nevertheless, given that solitary hunting wasps are also in need of the long-term metabolic alteration and developmental arrest of prey to provide fresh provisions to their progeny, a more versatile array of physiology-manipulating components is likely to be present in the venom of solitary wasps. Once these protein/peptide components with insecticidal or growth-regulating activity are identified, they can be exploited as alternative insect control agents, provided proper delivery protocols are established [140]. A spider venom neurotoxin (*Segestria florentina* toxin 1, SFI1) fused to the snowdrop lectin (*Galanthus nivalis* agglutinin, GNA) exhibited insecticidal activity against two homopteran sucking pests, the peach-potato aphid *Myzus persicae* and the rice brown planthopper *Nilaparvata lugens* [141], where the fusion protein gene can be employed for developing sucking pest-resistant transgenic crops. More recently, the ω-hexatoxin-Hv1a peptide (Hv1a), a neurotoxin from the Australian funnel web spider *Hadronyche versuta* acting on voltage-sensitive calcium channels, was fused to the carrier protein GNA to make Hv1a traverse the insect gut epithelium and access the central nervous system, thereby enhancing its oral toxicity [142,143]. In addition, recombinant baculoviruses expressing insect-selective toxins, hormones, or enzymes could enhance their insecticidal properties [144]. Once such neurotoxins or host/prey-regulatory molecules are identified and characterized from wasp venoms, similar biotechnical approaches can be attempted. Therefore, further research is needed for searching and characterizing wasp venom components with insecticidal and growth-regulating potential.

## 6. Concluding Remarks

Most studies on Hymenopteran venoms have been mainly focused on bees, parasitoid wasps, and social wasps. Possibly due to the limitation in sampling and venom collection, only very few previous studies have been conducted to identify and characterize the constituents of solitary wasp venoms. However, in view of diversity, ecology, and prey-specific behavior of solitary wasps, their venoms are still rich sources of novel bioactive substances. Recent introduction of cost-effective and high-throughput deep-sequencing technologies has enabled a rapid identification of genes encoding various venom peptides/proteins from venom gland transcriptomes. In addition, availability of highly sensitive mass spectrometry techniques allows for a more efficient proteomic/peptidomic analysis of a limited amount of solitary wasp venoms. A systematic and comparative analysis of venoms from solitary *vs.* social wasps would provide further insights into the venom evolution and phylogeny. Accumulation of functional data on bioactivity of various venom components would facilitate the application of wasp venoms for pharmacological, medical, and agricultural purposes.

## Appendix A

**Table A1:** Venom peptides of hunting wasps.

Sociality	Name	Sequence	Length (a.a)	Species	References
	***Neurotoxins***				
Solitary	α-PMTX	RIKIGLFQDLSKL	13	*Anoplius samariensis*	[23]
	β-PMTX	RIKIGLFQDLSRL	13	*Batozonellus maculifrons*	[23]
Social	AvTx-7	1210 Da (α-PMTX 1530 Da)	-	*Agelaia vicina*	[36]
	AvTx-8	1567 Da	-	*Agelaia vicina*	[35]
	***Kinins***				
Solitary	Bradykinin (BK)	RPPGFSPFR	9	mammal	-
	Megascoliakinin	RPPGFTPFRKA	11	*Megascolia flavifrons*	[145]
	Bradykinin	RPPGFSPFR	9	*Megacampsomeris prismatica*	[24]
	Thr^6^-BK	RPPGFTPFR	9	*Megacampsomeris prismatica*, *Campsomeriella annulata annulata*, *Carinoscolia melanosoma fascinate*, *Cyphononyx dorsalis*, *Megascolia flavifrons*, *Colpa interrupta*	[24,105,145]
Social	RA-Thr^6^-Bradykinin	RARPPGFTPFR	11	*Polybia paulista*	[40]
	RA-Thr^6^-Bradykinin-DT	RARPPGFTPFRDT	13	*Polybia paulista*	[40]
	Vespakinin-M	GRPHypGFSPFRID	14	*Vespa mandarinia*	[146]
	Vespakinin-X	ARPPGFSPFRIV	12	*Vespa xanthoptera*	[147]
	Vespakinin-A	GRPPGFSPFRVI	12	*Vespa analis*	[148]
	Vespakinin-AP **	ELPPGFTPFRII	12	*Vespa analis parallela*	[20]
	Vespakinin-T	GRPHypGFSPFRVI	12	*Vespa tropica*	[101]
	Vespakinin-C	KLPPGFTPFRII	12	*Vespa crabro flavogasciata*	[20]
	Vespulakinin	TAT(carbhy)T(carbhy)RRRGRPPGFSPFR	17	*(Para)Vespula maculifrons*	[149]
	Vespulakinin-L	TAR(NAcGal-Gal)TKRRGRPPGFSPFR	17	*Vespula lewisii*	[101]
	Polisteskinin 3	PyrTNKKKLRGRPPGFSPFR	18	*Polistes exclamans*, *Polistes annularis*, *Polistes fuscramatus*	[25]
	Polisteskinin-R	ARRPPGFTPFR	11	*Polistes rothneyi*	[25]
	Polisteskinin-J	RRRPPGFT(S)PFR	11	*Polistes jadwigae*	[101]
	Polisteskinin-C	SKRPPGFSPFR	11	*Polistes chnensis*	[101]
	PMM1	KRRPPGFTPFR	11	*Polistes major major*	[38]
	Protopolybiakinin-I	DKNKKPIRVGGRRPPGFTR	19	*Protopolybia exigua*	[39]
	Protopolybiakinin-II	DKNKKPIWMAGFPGFTPIR	19	*Protopolybia exigua*	[39]
	***Mastoparan-like Peptides***				
Solitary	EMP-AF	INLLKIAKGIIKSL-NH2	14	*Anterhynchium flavormarginatum micado*	[150]
	Eumenitin	LNLKGIFKKVASLLT	15	*Eumenes rubronotatus*	[29]
	EMP-OD (OdVP1)	GRILSFIKGLAEHL-NH2	14	*Orancistrocerus drewseni*	[30,66,114]
	OdVP3 ^a^	KDLHTVVSAILQAL-NH2	14	*Orancistrocerus drewseni*	[66,114]
	EpVP1 ^a^	INLKGLIKKVASLLT	15	*Eumenes pomiformis*	[18,114]
	EpVP2a ^a^	FDLLGLVKKVASAL-NH2	14	*Eumenes pomiformis*	[18,114]
	EpVP2b ^a^	FDLLGLVKSVVSAL-NH2	14	*Eumenes pomiformis*	[18,114]
Social	Mastoparan (MP)	INLKALAALAKKIL-NH2	14	*Vespula lewisii*	[110]
	Mastoparan-X	INWKGIAAMAKKLL-NH2	14	*Vespa xanthoptera*	[46]
	Mastoparan-A	IKWKAILDAVKKVL(I)-NH2	14	*Vespa analis*	[20,30]
	Mastoparan-B	LKLKSIVSWAKKVL-NH2	14	*Vespa basalis*	[30]
	Mastoparan-C	INW(L)KALLAVAKKIL-NH2	14	*Vespa crabro*	[20,30]
	Mastoparan-II	INLKALAALVKKVL-NH2	14	*Vespa orientalis*	[30]
	HR1	INLKAIAALVKKVL-NH2	14	*Vespa orientalis*	[37]
	Mastoparan-T1 *	INLKVFAALVKKFL-NH2	14	*Vespa tropica*	[19]
	Mastoparan-T2 *	INLKVFAALVKKLL-NH2	14	*Vespa tropica*	[19]
	Mastoparan-T3 *	INLRGFAALVKKFL-NH2	14	*Vespa tropica*	[19]
	Mastoparan-T4 *	INLFGFAALVKKFL-NH2	14	*Vespa tropica*	[19]
	protopolybia-MP I	INWLKLGKKVSAIL-NH2	14	*Protopolybia exigua*	[46]
	protopolybia-MP II	INWKAIIEAAKQAL-NH2	14	*Protopolybia exigua*	[46]
	protopolybia-MP III	INWLKLGKAVIDAL-NH2	14	*Protopolybia exigua*	[46]
	P-8	INWKALLDAAKKVL-NH2	14	*Protonectarina sylveirae*	[151]
	polybia-MP I	IDWKKLLDAAKQIL-NH2	14	*Polybia paulista*	[43]
	polybia-MP II	INWLKLGKMVIDAL-NH2	14	*Polybia paulista*	[42]
	polybia-MP III	IDWLKLGKMVMDVL-NH2	14	*Polybia paulista*	[42]
	polybia-MP IV	IDWLKLRVISVIDL-NH2	14	*Polybia paulista*	[40]
	polybia-MP V	INWHDIAIKNIDAL-NH2	14	*Polybia paulista*	[40]
	polybia-MP VI	IDWLKLGKMVM	11	*Polybia paulista*	[40]
	parapolybia-MP	INWKKMAATALKMI-NH2	14	*Parapolybia indica*	[46]
	parapolybia-MP	INWAKLGKLALEVI-NH2	14	*Parapolybia indica*	[30]
	Ropalidia-MP	INWAKLGKLALQAL-NH2	14	*Ropalidia*	[46]
	polistes-MP	VDWKKIGQHILSVL-NH2	14	*Polistes jadwigae*	[46]
	PMM2	INTKKIASIGKEVLKAL-NH2	17	*Polistes major major*	[38]
	Agelaia MP-I	INWLKLGKAIIDAL-NH2	14	*Agelaia pallipes pallipes*	[52]
	***Chemotactic Peptides***				
Solitary	OdVP2 (Orancis-protonectin)	ILGIITSLLKSL-NH2	12	*Orancistrocerus drewseni*	[30,66,114]
	EpVP6 ^b^	FGPVIGLLSGILKSLL	16	*Eumenes pomiformis*	[18,114]
	RbVP1 *^,b^	FLGGLIKGLVKAL-NH2	13	*Rhynchium brunneum*	[19]
Social	Protonectin	ILGTILGLLKGL-NH2	12	*Protonectarina sylveirae*, *Agelaia pallipes pallipes*	[52]
	Protonectin(1-6)	ILGTIL-NH2	6	*Agelaia pallipes pallipes*	[152]
	Paulista-CP (polybia-CP)	ILGTILGLLKSL-NH2	12	*Polybia paulista*	[43]
	Polybia-CP 2	ILGTILGKIL	10	*Polybia paulista*	[40]
	Polybia-CP 3	ILGTILGTFKSL-NH2	12	*Polybia paulista*	[40]
	Crabrolin	FLPLILRKIVTAL-NH2	13	*Vespa crabro*	[153]
	Ves-CP-T	FLPILGKILGGLL-NH2	13	*Vespa tropica*	P17231, [19]
	Ves-CP-T2 *	FLPIIGKLLSGLL-NH2	13	*Vespa tropica*	[19]
	Ves-CP-M	FLPIIGKLLSGLL-NH2	13	*Vespa mandarina*	P17232
	Ves-CP-A	FLPMIAKLLGGLL-NH2	13	*Vespa analis*, *Vespa analis parallela*	P17233, [20]
	Ves-CP-X	FLPIIAKLLGGLL-NH2	13	*Vespa xanthoptera*	P17234
	Ves-CP-L	FLPIIAKLVSGLL-NH2	13	*Vespula lewisi*	P17235
	VCP-5e	FLPIIAKLLGGLL-NH2	13	*Vespa magnifica*	[45]
	VCP-5f	FLPIPRPILLGLL-NH2	13	*Vespa magnifica*	[45]
	VCP-5g	FLIIRRPIVLGLL-NH2	13	*Vespa magnifica*	[45]
	VCP-5h	FLPIIGKLLSGLL-NH2	13	*Vespa magnifica*	[45]
	HP-1	LFRLIAKTLGSLM	13	*Vespa basalis*	[154]
	HP-2	LFRLLANTLGKIL	13	*Vespa basalis*	[154]
	HP-3	IFGLLAKTLGNLF	13	*Vespa basalis*	[154]
	HR2	FLPLILGKLVKGLL-NH2	14	*Vespa orientalis*	[37]
	PMM3	FLSALLGMLKNL-NH2	12	*Polistes major major*	[38]
	***Uncategorized Peptides***				
Solitary	Anoplin	GLLKRIKTLL-NH2	10	*Anoplius samariensis*	[27]
	Decoralin	SLLSLIRKLIT-NH2	11	*Oreumenes decoratus*	[28]
	OdVP4	LDPKVVQSLL-NH2	10	*Orancistrocerus drewseni*	[66,114]
	EpVP3	AINPKSVQSLL-NH2	11	*Eumenes pomiformis*	[18,114]
	EpVP3S	INPKSVQSLL-NH2	10	*Eumenes pomiformis*	[18,114]
	EpVP4a	LSPAVMASLA-NH2	10	*Eumenes pomiformis*	[18,114]
	EpVP4b	LSPAAMASLA-NH2	10	*Eumenes pomiformis*	[18,114]
	EpVP5	VHVPPICSHRECRK	14	*Eumenes pomiformis*	[18,114]
	As-peptide126	QDPPVVKMK-NH2	9	*Anoplius samariensis*	BAF65255
	Cd-125	DTARLKWH	8	*Cyphononyx dorsalis*	[107]
	Cd-146	SETGNTVTVKGFSPLR	16	*Cyphononyx dorsalis*	[107]
	EpDTX	IATICNLPIVSGNGQEEHIRWAYSIITHVCVSFRYTGKGGNRNNFFTERECRSYCYF	57	*Eumenes pomiformis*	[18,114]
	As-fr-19	VSFCLLPIVPGPCTQYVIRYAFQPSISACRRFTFGGCEGNDNNFMTRRDCEHYCEELL	58	*Anoplius samariensis*	[79]
Social	Vespin	CYQRRVAITAGGLKHRLMSSLIIIIIIRINYLRDNSVIILESSY	44	*Vespa magnifica*	[64]

* No name in the reference. Named in this review. ** Named the same with a previously known, different peptide. Renamed in this review. ^a^ Categorized as mastoparan-like peptides based on the sequence similarity with the previously reported venom peptides, without a mast cell degranulation activity test. ^b^ Categorized as chemotactic peptide-like peptides based on the sequence similarity with the previously reported venom peptides, without a chemotactic activity test.
